# The effects of plum products consumption on lipid profile in adults: A systematic review and dose–response meta‐analysis

**DOI:** 10.1002/fsn3.4000

**Published:** 2024-02-25

**Authors:** Azadeh Heydarian, Negin Tahvilian, Omid Asbaghi, Sahar Cheshmeh, Maryam Nadery, Naheed Aryaeian

**Affiliations:** ^1^ Student Research Committee, Faculty of Public Health Branch Iran University of Medical Sciences Tehran Iran; ^2^ Department of Nutrition, School of Public Health Iran University of Medical Sciences Tehran Iran; ^3^ Department of Nutrition, School of Public Health Shahid Sadoughi University of Medical Sciences Yazd Iran; ^4^ Nutrition and Food Security Research Center Shahid Sadoughi University of Medical Sciences Yazd Iran; ^5^ Cancer Research Center Shahid Beheshti University of Medical sciences Tehran Iran; ^6^ Student Research Committee Shahid Beheshti University of Medical Sciences Tehran Iran; ^7^ Molecular and Experimental Nutritional Medicine Department University of Potsdam Potsdam Germany; ^8^ Department of Dietetics and Nutrition, Robert Stempel College of Public Health & Social Work Florida International University Miami Florida USA

**Keywords:** lipid profile, meta‐analysis, plum, randomized controlled trial

## Abstract

Consumption of plum does not yet clearly affect the lipid profile. To ascertain the advantages of plum consumption on adult lipid profiles, we conducted a systematic review and meta‐analysis. We used pertinent keywords to search the databases of PubMed, Scopus, and ISI Web of Science up to November 10th, 2022, in order to find trials that were eligible. According to the analyses, eating plum significantly lowers LDL levels compared to controls (WMD: −12.50 mg/dL, 95% CI: −22.06, −2.94, *p* = .010). Although plum consumption did not result in significant changes in TG (WMD: 0.56 mg/dL, 95% CI: −6.02, 7.15, *p* = .866), TC (WMD: −12.35 mg/dL, 95% CI: −25.05, 0.37, *p* = .057), and HDL concentrations (WMD: −0.39 mg/dL, 95% CI: −4.69, 3.89, *p* = .855) compared to the control group. Intake of plums, particularly the intervention type of dried plums, significantly decreased TC levels in unhealthy subjects, according to subgroup analysis. The consumption of plums had a notably statistically significant effect on LDL levels when the intervention type was dried plum and unhealthy subjects were enrolled. Due to the very low to moderate quality of meta‐evidence, to show how eating plum improves lipid profile, further high‐quality research are still essential.

## Graphical abstract

Consumption of plums may reduce the level of TC and LDL in unhealthy people, but it has no effect on TG and HDL.

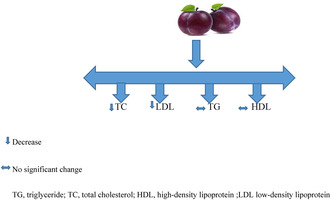

## 1 INTRODUCTION

Cardiovascular disease (CVD) is a leading cause of mortality worldwide (Hong et al., 2021). According to the European Society of Cardiology, approximately 113 million individuals in the 57 member countries experienced cardiovascular diseases (CVD) in 2019. These diseases were responsible for 45% of deaths in women and 39% of deaths in men (Timmis et al., 2022). CVD also contributed to 35.5% of all deaths in South Asia (Joseph et al., 2022). In about 36 percent of people with heart disorders, the risk factors of increased blood pressure, diabetes, and higher serum lipid levels are present (Stewart et al., 2020). Dyslipidemia refers to an elevated amount of serum TC, LDL‐C, and TG and a lower HDL‐C because of problems with lipoprotein metabolism. Dyslipidemia increases CVD and atherosclerosis‐related risk (Al‐Duais & Al‐Awthan, 2021). Cholesterol‐lowering medications are used to treat hyperlipidemia, but these medications' detrimental consequences include muscle weakness and liver damage and incur high healthcare costs (Chai et al., 2012). Plum belongs to the Rosaceae family, and its laxative properties are due to its high fiber content (Igwe & Charlton, 2016). Prunes are known to have been identified as having antioxidant properties. The high fiber content and various phytochemicals (Chlorogenic acids, Neochlorogenic, Cryptochlorogenic, Gallic acid, Caffeic acid, Quercetin, Cyanidin, Proanthocyanidins, and Delphidinin), Which are found in both dried plums and prune juice, benefits human health, including cancer, and CVD prevention (Stacewicz‐Sapuntzakis, 2013). Various plum products for consumption (dried plums, juice, essence, etc.) have been evaluated in studies, and different outcomes have been reported (Bhaswant et al., 2019; Chiu et al., 2017; Howarth et al., 2010). Studies suggest that Prunes contain antioxidant, anticancer, antidiabetic, cardioprotective, and neuroprotective effects (Chiu et al., 2017; Stacewicz‐Sapuntzakis, 2013). Plums contain high amounts of soluble and insoluble fiber, such as pectin and cellulose. Soluble fiber possibly reduces cholesterol absorption and increases bile excretion (Hong et al., 2021). Accordingly, it prevents the accumulation of cholesterol in the blood vessels and bile ducts (Walkowiak‐Tomczak, 2008). Another likely mechanism of plums' property of reducing serum TG is that plums' effect could increase the expression of PPARα mRNA. Activation of PPARα reduces Apoc‐III expression and induces lipoprotein lipase, decreasing serum TG (Utsunomiya et al., 2005). In obese, diabetic, and lean animal models, plum Ekisu (concentrated juice) and plum juice have improved lipid disorders (Tucakovic et al., 2018; Utsunomiya et al., 2005). In a study, taking dried plums at a dose of 50–100 g per day had beneficial effects on total cholesterol and HDL levels in postmenopausal women (Hong et al., 2021). In another study, Queen Garnet Plum Juice (QGPJ) consumption had no discernible impact on the level of the lipid profile (Noratto et al., 2015). Given that it was still unclear how plum modified plasma lipids, We opted to perform a comprehensive systematic review and meta‐analysis in order to gain further insights.

## 2 METHODS

This study utilized the Preferred Reporting Items for Systematic Reviews and Meta‐Analyses (PRISMA) methodology, which is a standardized approach for conducting systematic literature reviews and meta‐analyses (Moher, Liberati, Tetzlaff, Altman, & Group*, 2009) (File S1).

### 2.1 Search methodology

We conducted a comprehensive literature search using online databases., including PubMed, Scopus, and ISI Web of Science, up to November 10th, 2022, as a result of discovering related articles. We employed our search strategy, which comprises these keywords: (“black plums” OR “eugenia jambolana” OR “syzygium cumini” OR “jamun” OR “plums” OR “prunus salcina” OR “prunus damestica” OR “prunes” OR “prunes juice” OR “plum tree” OR “plum trees” OR “plum” OR “plums” OR “prunus cerasifera” OR “cherry plum tree” OR “cherry plum trees” OR “prunus salicina” OR “japanese plum” OR “japanese plums”) AND (Intervention OR “Intervention Study” OR “Intervention Studies” OR “controlled trial” OR randomized OR randomized OR random OR randomly OR placebo OR “clinical trial” OR Trial OR “randomized controlled trial” OR “randomized clinical trial” OR RCT OR blinded OR “double blind” OR “double blinded” OR trial OR “clinical trial” OR trials OR “Pragmatic Clinical Trial” OR “Cross‐Over Studies” OR “Cross‐Over” OR “Cross‐Over Study” OR parallel OR “parallel study” OR “parallel trial”). Our search was not limited by publication date or language. In order to ensure that all articles were cited, All relevant papers' citations were also verified.

### 2.2 Inclusion criteria

We took into account trials in our investigation if they fulfilled the following criteria: (1) Randomized controlled trials; (2) studies that recruited adults (≥18 years); (3) consisted of consuming plum; (4) RCTs lasting a minimum of 2 weeks of intervention duration; (5) Types of plum products (dried plums, juice, essence, and the rest…); (6) The studies assessed the serum lipid profile as a measure of the outcome for both the control and intervention categories.

### 2.3 Exclusion criteria

After reviewing the entire contents of the nominated publications, the following selection conditions were used to determine whether studies should be excluded from our meta‐analysis research: (1) Cohort, cross‐sectional, and case–control studies; (2) review articles; (3) ecological studies; and in the absence of a control group during examinations.

### 2.4 Data extraction

For each qualifying RCT, two separate researchers (AH and NT) completed the data extraction process. The following information was extracted for both the control and intervention groups: the name of the first author, the publication year, the study site, the study design, the sample size for each group, participant characteristics such as mean age, sex, and BMI, the study dosage and duration, and the mean changes and standard deviations (SDs) of lipid profile markers throughout the trial. If the data were provided in various units, we converted them to the unit that was most commonly used.

### 2.5 Quality assessment

Utilizing the Cochrane Collaboration's modified risk of bias methodology, which evaluates the likelihood of bias in seven different categories of RCTs, including random sequence generation to ensure proper randomization, allocation to verify if participant distribution was conducted by an unbiased and unaware person, reporting bias to assess if all relevant factors related to the object were reported, incomplete outcome data to check if both pre‐ and post measurements were reported, blinding of participants, personnel, and outcome assessors, as well as other bias sources, the quality of eligible publications was evaluated by examiners independently (AH and OA) (Higgins et al., 2011). As a result, adjectives like “Low,” “High,” or “Unclear” were employed to rate each domain. Additionally, the third author rectified any discrepancies.

### 2.6 Statistical analysis

Present examination, The DerSimonian and Laird approach was followed to collect the weighted mean differences (WMD) and standard deviations (SD) of TC, TG, LDL‐C, and HDL‐C between the plum and control conditions. These values were then used to calculate the total effect sizes using the random‐effects model. (DerSimonian & Laird, 1986). In cases where the provided mean changes were absent, we computed them using the specified formula: mean change = final values minus baseline values, and the changes in standard deviation (SD) were calculated using the following formula (Borenstein et al., 2011):

$$\mathrm{SD}\;\text{change}=\sqrt{\left[{\left(\mathrm{SD}\;\text{baseline}\right)}^{2}+{\left(\mathrm{SD}\;\text{final}\right)}^{2}-\left(2R\times \mathrm{SD}\;\text{baseline}\times \mathrm{SD}\;\text{final}\right)\right]}$$

The researchers converted the reported outcome variables (HDL‐C and LDL‐C, TG, and TC) from mmol/L to mg/dL using the existing applicable formulas. In addition, we utilized the Hozo et al. method to transform standard errors (SEs), 95% confidence intervals (CIs), and interquartile ranges (IQRs) into standard deviations (SDs) (Hozo et al., 2005). We used a random‐effects model, which takes into account variances between studies when determining the overall impact size. Additionally, we used the I‐square (*I* ^2^) statistic and Cochran's Q test to examine between‐study heterogeneity (*I* ^2^) (Higgins et al., 2003). High between‐study heterogeneity was defined as *I* ^2^ > 40% or *p*‐value < .05. To identify potential heterogeneity sources (Higgins & Thompson, 2002). Study duration (≤8 and >8 weeks), Intervention type dried plum (prunes), Queen Garnet (QG) plums, prune essence concentrates (PEC), baseline serum levels of the lipid profile, and Health status (healthy and unhealthy) were the pre‐planned parameters that were used to create subgroup analyses. The study utilized fractional polynomial modeling to analyze the potential non‐linear impacts of treatment duration (measured in weeks). In addition, we employed meta‐regression analysis to differentiate between confounding variables and the linear associations among effectiveness size, number of samples, and intervention length (Mitchell, 2012). A sensitivity analysis was performed to establish how each individual study affected the total estimation (Tobias, 1999). The visually inspected funnel plot test and Egger's regression analysis were performed to evaluate the probability of publication bias (Egger et al., 1997). STATA version 11.2 was used for the quantitative evaluation (Stata Corp, College Station, TX). *p*‐Values less than .05 were regarded in all analyses as statistically significant.

### 2.7 Certainty assessment

The overall level of certainty in the evidence from all the studies was assessed using the guidelines provided by the GRADE (Grading of Recommendations Assessment, Development, and Evaluation) Working Group. The assessment parameters were used to categorize the value of the findings into four distinct categories: high, moderate, low, and seriously poor. (Guyatt et al., 2008).

## 3 RESULTS

### 3.1 Study selection

**FIGURE 1:**
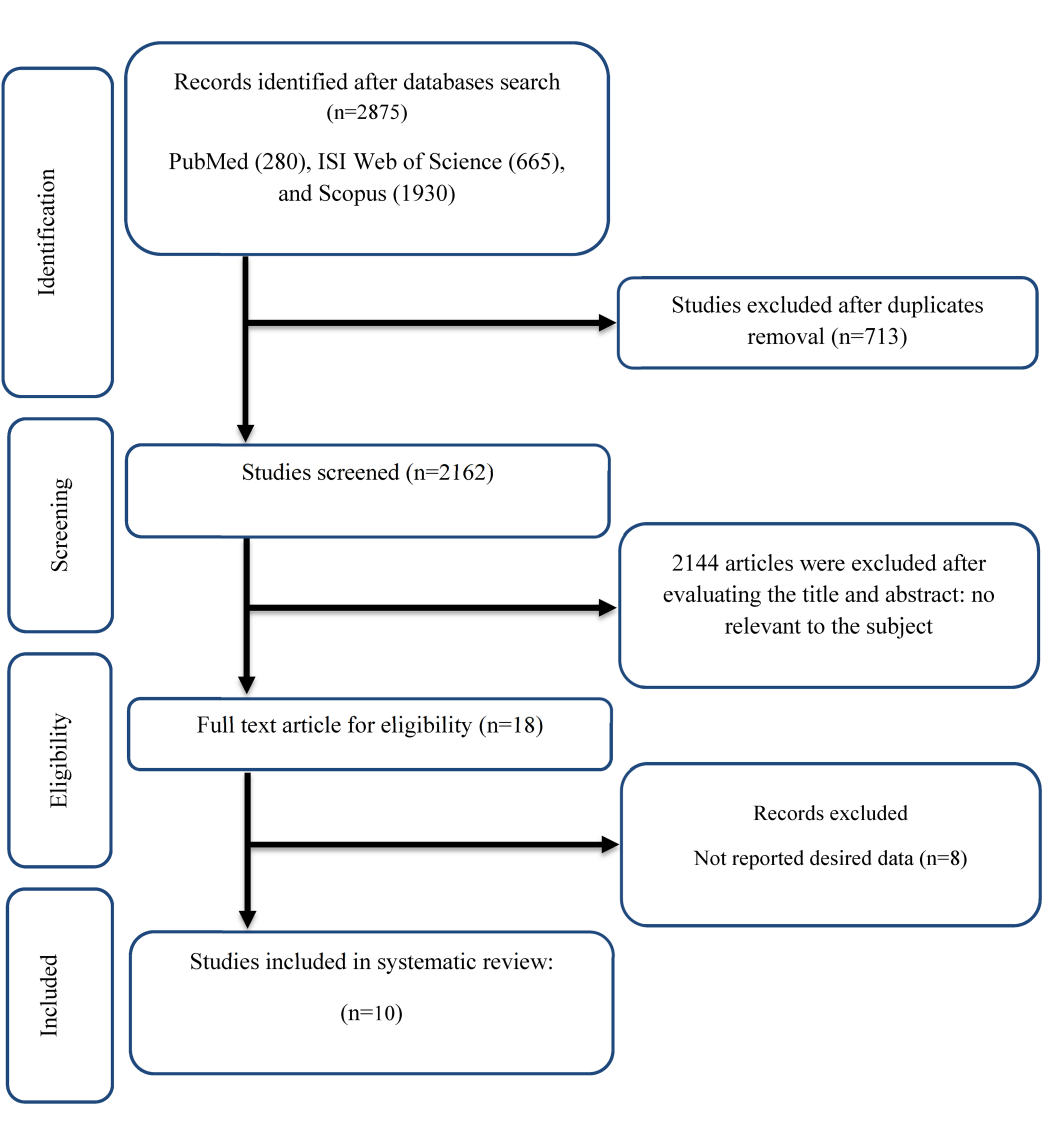
Flow chart of study selection for inclusion trials in the systematic review.

Initially looking, we found 2875 records; 713 duplicates were found and eliminated. 18 papers were kept for additional review after being first filtered according to the title and summary. Due to the requirement that the articles fit the inclusion criteria, eight were excluded. Subsequently, 10 trials that met the inclusion criteria were considered in the amalgamation of qualitative and quantitative data (meta‐analysis) (Figure 1) (Ahmed et al., 2010; Bhaswant et al., 2019; Chai et al., 2012; Chiu et al., 2017; Clayton et al., 2019; Hong et al., 2021; Howarth et al., 2010; Santhakumar et al., 2015; Tinker et al., 1991; Tucakovic et al., 2018).

### 3.2 2 the listed studies' characteristics

Table 1 lists the general features of the studies that were considered. The research papers were released between 1991 and 2021 and were performed in the USA (Chiu et al., 2017; Clayton et al., 2019; Hong et al., 2021; Howarth et al., 2010; Tinker et al., 1991), Pakistan (Ahmed et al., 2010), Australia (Bhaswant et al., 2019; Santhakumar et al., 2015; Tucakovic et al., 2018), and Taiwan (Chiu et al., 2017). The suggested daily dosage of plum ranged from 23 to 250 g, and the duration of follow‐up spanned 2–24 weeks. Seven studies (Ahmed et al., 2010; Bhaswant et al., 2019; Chiu et al., 2017; Clayton et al., 2019; Santhakumar et al., 2015; Tinker et al., 1991; Tucakovic et al., 2018) conducted on both sexes and others on females (Chai et al., 2012; Hong et al., 2021; Howarth et al., 2010). The trials encompassed samples varying in magnitude from 13 to 100 participants. A total of 562 individuals took part in these studies, with 315 being allocated to the plum supplementation group and 247 to the control group. The individuals involved, who ranged in age from 18 to 71 on average, included patients with Mild hypercholesterolemia (Bhaswant et al., 2019; Chiu et al., 2017; Tinker et al., 1991), Pre‐hypertensive adult patients (Ahmed et al., 2010), and healthy subjects (Chai et al., 2012; Clayton et al., 2019; Hong et al., 2021; Howarth et al., 2010; Santhakumar et al., 2015; Tucakovic et al., 2018) (Table 1). Furthermore, the risk of bias assessment is presented in Table 2.

**TABLE 1:** Characteristics of the included studies.

Studies	Country	Study design	Participant	Sex	Sample size		Trial duration (week)	Means age		Means BMI		Intervention		
					IG	CG		IG	CG	IG	CG	Prune type	Prune dose (g/day)	Control group
Tinker et al. (1991)	USA	Crossover	Mild hypercholesterolemic	M/F	41	41	4	46.5 ± 12.4	46.5 ± 12.4	NR	NR	Prunes	100	Grape juice
Howarth et al. (2010)	USA	Crossover	Healthy	F	26	26	2	36.4 ± 9.2	36.4 ± 9.2	NR	NR	Dried plum	90	Low fat cookie
Ahmed et al. (2010)	Pakistan	R	Pre‐hypertensive adult patients	M/F	31	16	8	49 ± 9	42 ± 10	NR	NR	Dried plum in water	11.5	Water
Ahmed et al. (2010)	Pakistan	R	Pre‐hypertensive adult patients	M/F	24	17	8	42 ± 12	42 ± 10	NR	NR	Dried plum in water	23	Water
Chai et al. (2012)	USA	R	Healthy postmenopausal women	F	55	45	12	57.5 ± 4.01	55.6 ± 5	24.9 ± 4.6	24.8 ± 4.1	Dried plum	75	Dried apple 100 gr
Santhakumar et al. (2015)	Australia	R, DB, crossover	Healthy	M/F	13	13	4	30 ± 3	30 ± 3	NR	NR	Queen Garnet plum juice	200 mL	Placebo juice
Chiu et al. (2017)	Taiwan	R, PC, CO	Mild hypercholesterolemic	F:33 M: 27	20	10	4	18–53	18–53	21.57 ± 2.45	23.59 ± 4.04	Prune essence concentrates	50 mL	50 mL of simulated prune drink
Chiu et al. (2017)	Taiwan	R, PC, CO	Mild hypercholesterolemic	F:33 M: 27	20	10	4	18–53	18–53	22.82 ± 2.98	23.59 ± 4.04	Prune essence concentrates	100 mL	50 mL of simulated prune drink
Tucakovic et al. (2018)	Australia	R, DB, PC, crossover	Healthy	F: 10 M:11	20	20	4	18–65	18–65	24.61 ± 2.64	24.34 ± 2.54	Queen Garnet plum juice	200 mL/day	Placebo drink
Clayton et al. (2019)	USA	Parallel	Healthy overweight adult	F: 30 M: 19	24	21	8	35.3 ± 12	39.6 ± 11.1	33.1 ± 31.8	29.1 ± 16.1	Dried plum	83.6	Low fat muffin
Bhaswant et al. (2019)	Australia	R, DB	Mild hypercholesterolemic	F: 14 M:15	14	15	12	47 ± 11	38 ± 14	NR	NR	Queen Garnet plums juice	250 mL/day	Raspberry cordial
Hong et al. (2021)	USA	Parallel, CO	Postmenopausal women	F	14	7	24	68.5 ± 4.3	71 ± 2.9	24.8 ± 3.9	25 ± 4.3	Dried plum	50	No intervention
Hong et al. (2021)	USA	Parallel, CO	Postmenopausal women	F	13	6	24	70.4 ± 3.7	71 ± 2.9	24.9 ± 4	25 ± 4.3	Dried plum	100	No intervention

Abbreviations: BMI, body mass index; CG, control group; CO, controlled; DB, double blind; F, female; IG, intervention group; M, male; NR, not reported; PC, placebo‐controlled; R, randomized.

**TABLE 2:** Risk of bias assessment.

Studies	Random sequence generation	Allocation concealment	Selective reporting	Other sources of bias	Blinding (participants and personnel)	Blinding (outcome assessment)	Incomplete outcome data	General risk of bias
Tinker et al. (1991)	L	L	L	L	H	U	L	L
Howarth et al. (2010)	L	L	H	L	H	U	L	M
Ahmed et al. (2010)	L	L	L	H	H	U	L	M
Chai et al. (2012)	L	L	H	L	H	U	L	M
Santhakumar et al. (2015)	L	L	L	H	L	U	L	L
Chiu et al. (2017)	L	L	L	H	H	U	L	M
Tucakovic et al. (2018)	L	L	L	L	L	U	L	L
Clayton et al. (2019)	L	L	L	L	H	U	L	L
Bhaswant et al. (2019)	L	L	L	H	L	U	L	L
Hong et al. (2021)	L	L	L	H	H	U	L	M

*Note*: General Low Risk < 2 high risk, General moderate risk = 2 high risk, General high risk > 2 high risk.

Abbreviations: H, High; L, Low; M, Moderate; U, Unclear.

### 3.3 Effect of plum supplementation on TG

**FIGURE 2:**
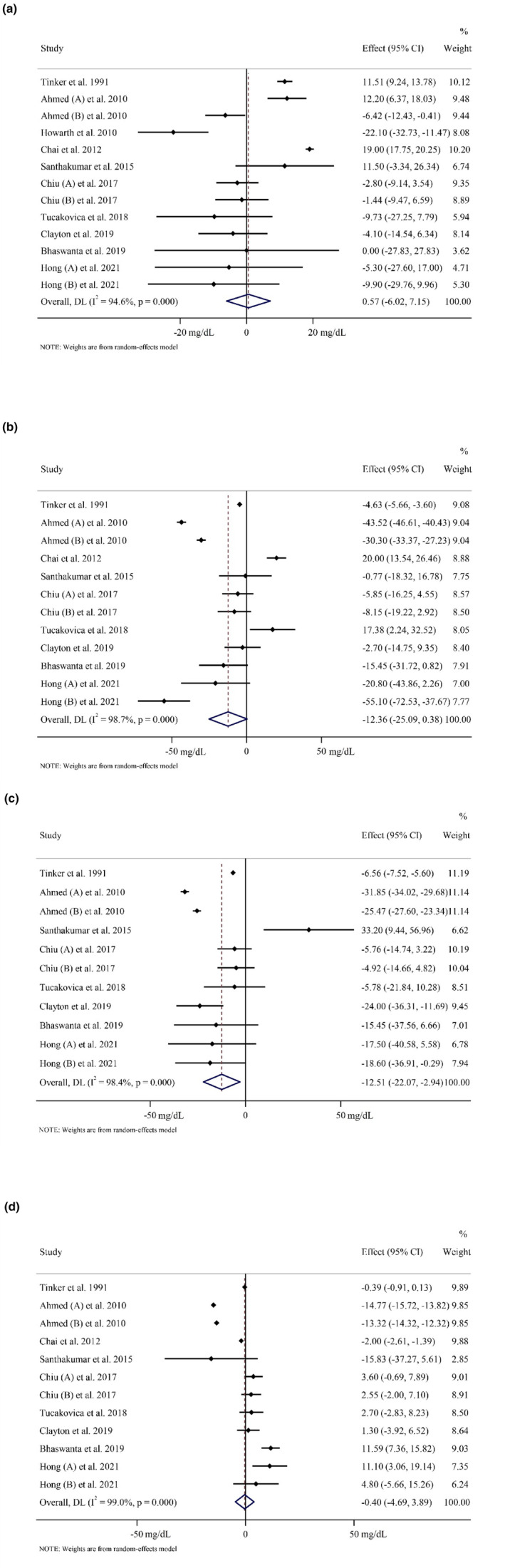
Forest plot detailing weighted mean difference and 95% confidence intervals (CIs) for the effect of plum consumption on (a) TG (mg/dL); (b) TC (mg/dL); (c) LDL (mg/dL); (d) HDL (mg/dL).

TG was reported as an outcome measure in ten studies with 562 participants (case = 315 and control = 247) and 13 effect sizes. The random‐effects model's overall findings revealed that eating plums had no influence on TG levels (WMD: 0.56 mg/dL, 95% CI: −6.02, 7.15, *p* = .866). Between‐study heterogeneity was noted (*I* ^2^ = 94.6%, *p* < .001) (Figure 2a). In addition, subgroup analysis revealed the same results (Table 3).

**TABLE 3:** Subgroup analyses of prune consumption on lipid profile in adults.

	Number of studies	WMD (95%CI)	*p*‐Value	Heterogeneity	
				P heterogeneity	*I* ^2^
Subgroup analyses of prune consumption on TG					
Overall effect	13	0.56 (−6.02, 7.15)	.866	<0.001	94.6%
Baseline TG (mg/dL)					
<150	11	0.09 (−7.00, 7.20)	.979	<0.001	94.2%
≥150	2	2.90 (−15.34, 21.15)	.755	<0.001	94.7%
Trial duration (week)					
≤8	9	−0.78 (−8.28, 6.72)	.839	<0.001	90.9%
>8	4	2.86 (−15.40, 21.13)	.759	0.002	79.1%
Health status					
Unhealthy	6	2.72 (−5.09, 10.54)	.495	<0.001	90.0%
Healthy	7	−2.50 (−18.31, 13.30)	.756	<0.001	93.8%
Intervention type					
1	8	1.32 (−6.40, 9.06)	.736	<0.001	95.8%
2	3	1.44 (−12.71, 15.60)	.842	0.191	39.6%
3	2	−2.27 (−7.25, 2.69)	.370	0.794	0.0%
Subgroup analyses of prune consumption on TC					
Overall effect	12	−12.35 (−25.05, 0.37)	.057	<0.001	98.7%
Baseline TC (mg/dL)					
≥200	2	−6.86 (−15.45, 1.72)	.117	0.193	40.9%
<200	10	−12.91 (−28.15, 2.32)	.097	<0.001	97.8%
Trial duration (week)					
≤8	8	−10.49 (−25.77, 4.79)	.179	<0.001	99.1%
>8	4	−17.32 (−53.92, 19.28)	.354	<0.001	96.1%
Health status					
Unhealthy	6	−18.23 (−35.95, −0.51)	**.044**	<0.001	99.3%
Healthy	6	−6.21 (−27.49, 15.06)	.567	<0.001	93.3%
Intervention type					
1	7	−19.09 (−36.38, −1.79)	**.030**	<0.001	99.3%
2	3	0.58 (−18.76, 19.93)	.953	0.015	76.3%
3	2	−6.92 (−14.51, 0.65)	.073	0.767	0.0%
Subgroup analyses of prune consumption on LDL					
Overall effect	11	−12.50 (−22.06, −2.94)	**.010**	<0.001	98.4%
Baseline LDL (mg/dL)					
≥100	6	−13.96 (−28.63, 0.69)	.062	<0.001	98.9%
<100	5	−10.50 (−25.17, 4.17)	.161	<0.001	90.1%
Trial duration (week)					
≤8	8	−11.16 (−22.05, −0.26)	**.045**	<0.001	98.9%
>8	3	−17.36 (−29.40, −5.33)	**.005**	0.977	0.0%
Health status					
Unhealthy	6	−15.28 (−27.63, −2.94)	**.015**	<0.001	99.2%
Healthy	5	−7.82 (−25.23, 9.57)	.378	0.001	78.8%
Intervention type					
1	6	−20.90 (−33.71, −8.08)	**.001**	<0.001	99.2%
2	3	3.28 (−22.98, 29.55)	.807	0.007	79.7%
3	2	−5.37 (−11.97, 1.22)	.111	0.901	0.0%
Subgroup analyses of prune consumption on HDL					
Overall effect	12	−0.39 (−4.69, 3.89)	.855	<0.001	99.0%
Baseline HDL (mg/dL)					
≥50	6	−0.19 (−1.75, 1.36)	.805	<0.001	80.8%
<50	6	−1.02 (−6.87, 4.82)	.731	<0.001	97.7%
Trial duration (week)					
≤8	8	−3.56 (−10.10, 2.98)	.286	<0.001	99.3%
>8	4	6.17 (−3.07, 15.41)	.191	<0.001	94.0%
Health status					
Unhealthy	6	−1.99 (−9.40, 5.41)	.598	<0.001	99.5%
Healthy	6	1.82 (−2.57, 6.23)	.416	0.004	71.2%
Intervention type					
1	7	−2.89 (−8.30, 2.50)	.293	<0.001	99.4%
2	3	3.89 (−5.94, 13.73)	.438	0.004	81.8%
3	2	3.10 (−0.01, 6.22)	.051	0.742	0.0%

*Note*: Intervention type 1: dried plum (prunes); 2: Queen Garnet plums; 3: prune essence concentrates.

Abbreviations: CI, confidence interval; HDL, High‐density lipoprotein; LDL, Low‐density lipoprotein; TC, Total Cholesterol; TG, Triglycerides; WMD, weighted mean differences.

*p*‐value ≤ 0.05 is considered statically significant.

### 3.4 Effect of plum supplementation on TC concentrations

Pooling effect sizes from 9 publications with 12 effect sizes, including 510 participants (case = 289, and control = 221), we found that plum intake had no significant effect on TC concentrations compared (WMD: −12.35 mg/dL, 95% CI: −25.05, 0.37, *p* = .057), with considerable between‐study heterogeneity (*I* ^2^ = 98.7%, *p* < .001) (Figure 2b). Subgroup analysis revealed that eating plums significantly reduced TC levels in those who were unhealthy and when the intervention type was dried plum (Table 3).

### 3.5 LDL content and the response to plum supplementation

In 11 studies, 410 subjects (case = 234 and control = 176) were examined to ascertain plum consumption's effects on LDL levels. The combined estimations showed that those who took plum supplements had significantly lower LDL concentrations than those who did not (WMD: −12.50 mg/dL, 95% CI: −22.06, −2.94, *p* = .010). There was a significant amount of variability between studies (*I* ^2^ = 98.4%, *p* < .001) (Figure 2c). Subgroup analysis revealed that when intervention type dried plum and unhealthy subjects were recruited, the plum intake significantly affected LDL levels (Table 3).

### 3.6 Effectiveness of supplementing with plums on HDL levels

Overall, the impact of plum consumption on HDL levels was investigated in nine eligible studies with 12 effect sizes, and 510 participants (case = 289 and control = 221). Combining their results using a random‐effects model, our findings indicate that there was no significant alteration in HDL concentrations after the intervention, when compared to the control group. (WMD: −0.39 mg/dL, 95% CI: −4.69, 3.89, *p* = .855) with significant between‐study heterogeneity (*I* ^2^ = 99.0%, *p* < .001) (Figure 2d). Subgroup analysis produced no different findings (Table 3).

### 3.7 Publication bias

**FIGURE 3:**
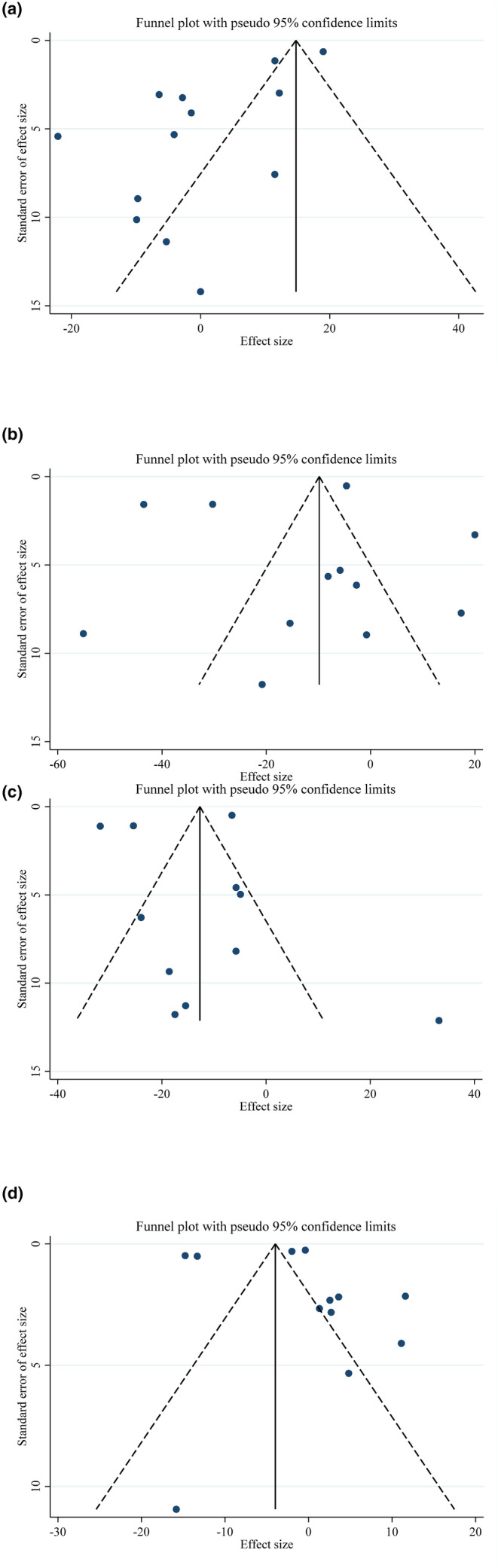
Funnel plots for the effect of plum consumption on (a) TG (mg/dL); (b) TC (mg/dL); (c) LDL (mg/dL); (d) HDL (mg/dL).

In the meta‐analysis examining eating plum's impact on TG (*p* = .50), TC (*p* = .53), LDL (*p* = .75), and HDL (*p* = .15), there was no indication of reporting bias when using Begg's test to assess publication bias. There was, however, a substantial publication bias for TG (*p* = .001), but not for TC (*p* = .55), LDL (*p* = .59), or HDL (*p* = .95), based on Egger's regression test, a visual inspection of the funnel plot revealed a leftward asymmetry for TG and a symmetrical distribution for other factors. (Figure 3a–d).

### 3.8 Sensitivity analysis

There is no evidence of sensitivity for TG and HDL. However, after removing studies by Chai et al. (2012) (WMD: −15.51 mg/dL, 95% CI: −28.74, −2.28), Santhakumar et al. (2015) (WMD: −13.33 mg/dL, 95% CI: −26.66, −0.00), and Tucakovic et al. (2018) (WMD: −14.96 mg/dL, 95% CI: −28.25, −1.67), the overall effect of plum intake on TG level was significantly changed. Also, after the elimination of Ahmed's study (Ahmed et al., 2010) (B), the effect of plum consumption on LDL levels was changed to insignificant. (WMD: −10.68 mg/dL, 95% CI: −21.96, 0.59).

### 3.9 Non‐linear and linear dose responses between duration of plum intake and lipid profile

**FIGURE 4:**
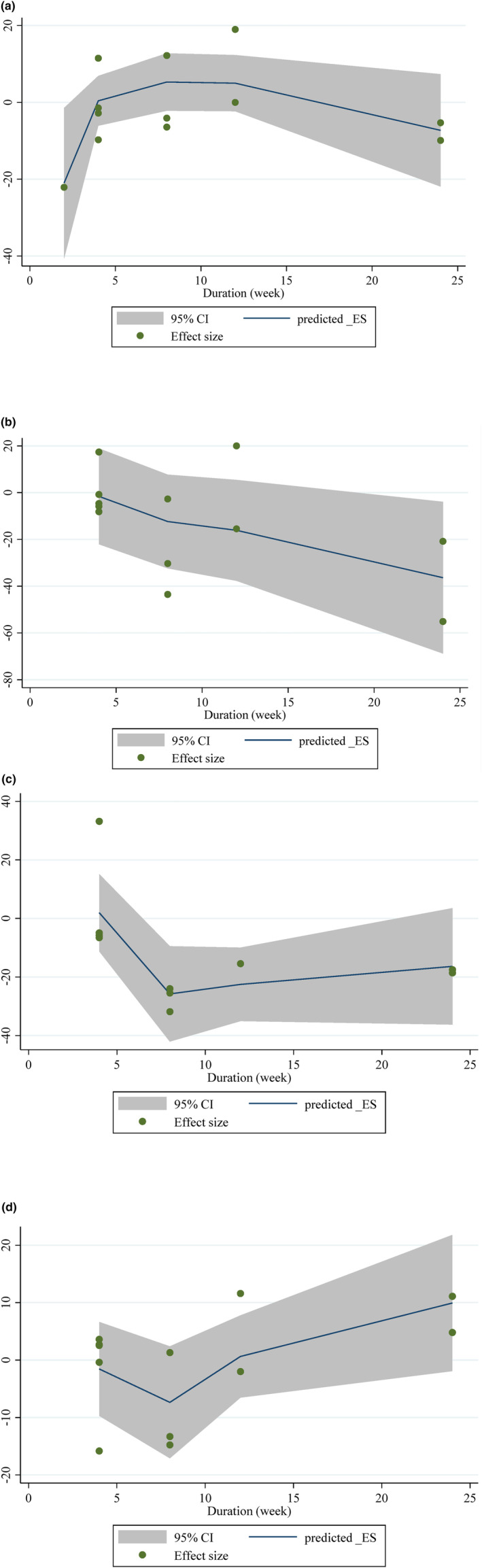
Non‐linear dose–response relations between prune consumption and absolute mean differences. Dose–response relations between duration of intervention (week) and absolute mean differences in (a) TG (mg/dL); (b) TC (mg/dL); (c) LDL (mg/dL); (d) HDL (mg/dL).

Plum intake changed HDL significantly based on the duration of the intervention (*r* = −18.71, P‐nonlinearity = 0.045) in a non‐linear fashion. Additionally, significant associations with other outcomes in non‐linear dosage patterns were not observed (Figure 4a–d).

**FIGURE 5:**
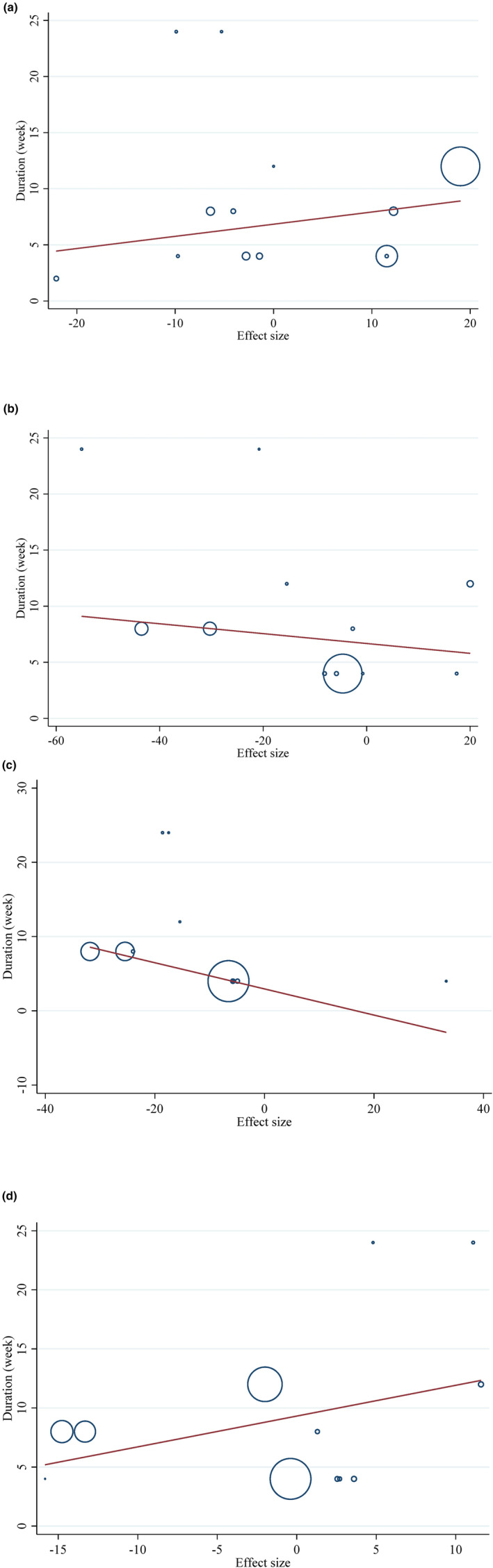
Linear dose–response relations between prune consumption and absolute mean differences. Dose–response relations between duration of intervention (week) and absolute mean differences in (a) TG (mg/dL); (b) TC (mg/dL); (c) LDL (mg/dL); (d) HDL (mg/dL). Each circle is a study and the size of each circle shows its effect size.

The potential relationship between a change in lipid profile and the length of the intervention was investigated using meta‐regression using the random‐effects model. Nevertheless, a notable linear correlation was observed between the duration of the intervention and the alterations in LDL levels. (Figure 5a–d).

### 3.10 Grading of evidence

The GRADE methodology was employed to assess the level of certainty of the evidence (Table 4) and concluded that the evidence for LDL‐C is of moderate quality due to significant inconsistency, while the evidence for TC and HDL is of low quality due to serious imprecision and inconsistency. Nevertheless, the evidence supporting TG was deemed of poor quality as a result of its significant variability, imprecision, and publication bias.

**TABLE 4:** GRADE profile of prune consumption for lipid profile.

Outcomes	Risk of bias	Inconsistency	Indirectness	Imprecision	Publication bias	Number of intervention/control	Quality of evidence
TG	No serious limitation	Serious limitation^a^	No serious limitation	Serious limitation^b^	Serious limitation^c^	562 (315/247)	⊕◯◯◯ Very Low
TC	No serious limitation	Serious limitation^a^	No serious limitation	Serious limitation^b^	No serious limitation	510 (289/221)	⊕⊕◯◯ Low
LDL	No serious limitation	Serious limitation^a^	No serious limitation	No serious limitation	No serious limitation	410 (234/176)	⊕⊕⊕◯ Moderate
HDL	No serious limitation	Serious limitation^a^	No serious limitation	Serious limitation^b^	No serious limitation	510 (289/221)	⊕⊕◯◯ Low

^a^ There is significant heterogeneity for TG (*I* ^2^ = 94.6%), TC (*I* ^2^ = 98.7%), LDL‐C (*I* ^2^ = 98.4%), and HDL‐C (*I* ^2^ = 99.0%).

^b^ There is no evidence of significant effects of prune consumption on TG, TC, and HDL‐C.

^c^ There is significant publication bias for TG (*p* = .001).

## 4 DISCUSSION

Prunes are rich in polyphenols and have antioxidant activity; previous in vitro and in vivo data showed a decrease in oxidative stress, anti‐inflammatory effects, and lipid profile improvement from prunes (Bu et al., 2009; Donovan et al., 1998; Gallaher & Gallaher, 2008; Kumar et al., 2009; Lucas et al., 2000). These positive results led to the first human clinical trial studies that looked at how plums affected people's lipid profiles. In this way, this systematic review and meta‐analysis was intended to determine how eating plum could influence the lipid profiles of adults. The qualitative and quantitative analysis of the 10 studies that were eligible for this study showed that eating plum did not change the levels of TG, TC, or HDL‐C. However, LDL‐C concentrations decreased significantly compared to the control.

Subgroup analysis revealed that the consumption of plums had a significant effect on lowering total cholesterol (TC) levels in individuals with poor health and when the intervention involved dried plums. When the intervention type was dried plum and unhealthy participants were recruited, plum consumption significantly affected LDL levels. Tucakovic et al. demonstrated that consumption of Queen Garnet Plum Juice (QGPJ) did not alter the lipid profile level in healthy subjects (Tucakovic et al., 2018). In line with our findings, a previous study found that eating 100 g of prunes daily for 6 weeks reduced LDL‐C and TC levels in patients with moderate hypercholesterolemia. In addition, there were no notable alterations in the levels of HDL or TG; this study lacked a control group (Walkowiak‐Tomczak et al., 2018). Similarly, According to a cross‐over design study by Tinker et al., men with mild hypercholesterolemia who consumed 12 prunes per day for 8 weeks saw an increase in fiber intake and a decrease in LDL and total cholesterol levels. However, there were no discernible improvements in HDL and triglyceride levels (Tinker et al., 1991). The results of a randomized, crossover study design with 2 weeks of washout showed that healthy postmenopausal women who consumed six prunes (dried plum) (approximately 42 g) or two prunes (approximately 14 g) daily for 2 weeks had no significant changes in total cholesterol, LDL‐C, HDL‐C, or TG during the study protocol. In this study, the low duration of prune intake and the normal baseline LDL‐C levels of participants have been mentioned as the reasons for no significant improvements in LDL (Al‐Dashti et al., 2019). A previous study found that giving 12 healthy people 450 g of plums and 530 g of cherries simultaneously for 2 weeks reduced serum TC concentrations significantly. However, no obvious differences were discovered in LDL‐cholesterol, TG, or HDL‐C (Sung et al., 2005). Howarth et al. (2010) reported that taking dried plums as a snack can help reduce total fat consumption and increase fiber. Triglyceride levels did not change in this study with dried plum consumption. The findings from an animal study indicated that, in normal rats, both Prunus divaricata freeze‐dried juice (PFDJ) powder (200, 400, and 800 mg/kg) and Prunus divaricata freeze‐dried hydroalcoholic extract (PFDE) powder (100, 200, and 400 mg/kg) were not adequate to diminish serum TC, LDL‐cholesterol TG, and improved HDL‐C levels. However, all test doses of PFDJ (200, 400, and 800 mg/kg) and two higher doses of PFDE (200, 400 mg/kg, but not 100 mg/kg) were able to significantly lower serum TG levels in diabetic rats. During the treatment's subacute phase (30th day), only the PFDJ fraction helped lower TC (200, 400, and 800 mg/kg) and LDL‐C (400, 800 mg/kg, but not 200 mg/kg). In the diabetic group, PFDJ (400, 800 mg/kg, not 200 mg/kg) likewise raised HDL‐C levels (Minaiyan et al., 2014). Although the effects of prune on TC, TG, and HDL were insignificant, the effects of prune components such as chlorogenic acid and Polyphenols have been observed in improving lipid profiles (Potì et al., 2019; Stacewicz‐Sapuntzakis, 2013; Wang et al., 2019). Even with appropriate food consumption, individuals with CVD risk factors have been shown to have lower levels of nutrients and antioxidants than healthy subjects in earlier research (Godala et al., 2017; Hadi et al., 2020). Thus, it is not too far from the truth if adding sources of antioxidants has a better effect on TC and LDL‐C in people who are not healthy than in healthy people. The amount of total cholesterol (TC) in the blood decreased when people ate more fiber. Fiber and antioxidant components in plums can promote the elimination of leftover cholesterol in the blood, which is helped by bile (Trimurtini et al., 2021; Walkowiak‐Tomczak, 2008). An in vitro study revealed that, compared to the prune juice, the prune extract was a more potent inhibitor of LDL oxidation, and both strongly prevented LDL oxidation in the plasma of healthy participants (Donovan et al., 1998). The lower risk of serious CVD events was significantly positive correlation with lowering LDL‐C, according to the prior study. A 1 mmol/L reduction in LDL‐C levels was associated with a 21 percent decrease in the prevalence of vascular events (Shimizu et al., 2015). An inflammatory response causes LDL oxidation in the arterial walls, and macrophages engulf the oxidized LDL and become foam cells, causing plaque formation. For atherosclerosis to be less common, LDL oxidation and plaque development may need to be prevented. Contrarily, the fiber and polyphenols in prunes may alter the composition of the gut's microbial population by boosting the growth of prebiotics and probiotics, which reduce inflammation while increasing the production of short‐chain fatty acids (SCFAs) and decreasing the levels of lipopolysaccharides (LPS). These modifications may lessen the development of atherosclerotic plaque (Foley, 2020; Stacewicz‐Sapuntzakis et al., 2001).

### 4.1 Strengths and limitations

An important advantage of this systematic review and meta‐analysis was its thorough examination of the impact of consuming plums on lipid profile indices (total cholesterol, low‐density lipoprotein, high‐density lipoprotein, and triglycerides) in adults, which had not been done before. Simultaneously evaluating the effects of all four indices allows us to gain a comprehensive understanding of the actual impact of prune consumption on the lipid profile. Furthermore, we used meta‐regression to distinguish confounders and linear relationships between effect size and sample size, and intervention duration. There are some limitations and drawbacks to be addressed in the current meta‐analysis study. The absence of high‐quality trials is the major downside of this meta‐analysis. Also, there was considerable heterogeneity in the results of the investigations included. Several studies that were part of this meta‐analysis used different types of plum products, placebos, and clinical conditions. Thus, it is impossible to reach clear conclusions in this field, and more research is needed.

## 5 CONCLUSION

In conclusion, plum consumption, especially the intervention type of dried plum, may improve lipid profiles by decreasing TC levels in unhealthy subjects and also decreasing LDL levels statically. However, it does not have to affect TG and HDL. Additionally, well‐designed RCTs with longer interventions and bigger sample sizes are necessary to support the effects of plum on lipid profiles.

## AUTHOR CONTRIBUTIONS

**Azadeh Heydarian:** Data curation (equal); investigation (equal); methodology (equal); writing – original draft (equal); writing – review and editing (equal). **Negin Tahvilian:** Data curation (equal); investigation (equal); methodology (equal); writing – original draft (equal); writing – review and editing (equal). **Omid Asbaghi:** Formal analysis (equal); software (equal); validation (equal). **Sahar Cheshmeh:** Writing – review and editing (equal). **Maryam Nadery:** Writing – review and editing (equal). **Naheed Aryaeian:** Project administration (equal); supervision (equal); validation (equal); writing – review and editing (equal).

## FUNDING INFORMATION

This research was supported by grant No 1401‐3‐15‐24,756 from Iran University of Medical Sciences.

## CONFLICT OF INTEREST STATEMENT

The authors declared that there is no conflicts of interest.

## Supporting information


**File S1.**:

**File S2.**.

## Data Availability

On reasonable request, the corresponding author will provide the datasets created and used in the current study.

## References

[fsn34000-bib-0001] Ahmed, T. , Sadia, H. , Batool, S. , Janjua, A. , & Shuja, F. (2010). Use of prunes as a control of hypertension. Journal of Ayub Medical College, Abbottabad, 22(1), 28–31. 21409897

[fsn34000-bib-0002] Al‐Dashti, Y. A. , Holt, R. R. , Carson, J. G. , Keen, C. L. , & Hackman, R. M. (2019). Effects of short‐term dried plum (prune) intake on markers of bone resorption and vascular function in healthy postmenopausal women: A randomized crossover trial. Journal of Medicinal Food, 22(10), 982–992. 31194598 10.1089/jmf.2018.0209

[fsn34000-bib-0003] Al‐Duais, M. A. , & Al‐Awthan, Y. S. (2021). Khat chewing and lipid profile in human and experimental animals. BioMed Research International, 2021, 6001885. 34977243 10.1155/2021/6001885 PMC8719995

[fsn34000-bib-0004] Bhaswant, M. , Brown, L. , & Mathai, M. L. (2019). Queen garnet plum juice and raspberry cordial in mildly hypertensive obese or overweight subjects: A randomized, double‐blind study. Journal of Functional Foods, 56, 119–126.

[fsn34000-bib-0005] Borenstein, M. , Hedges, L. , Higgins, J. , & Rothstein, H. (2011). Introduction to meta‐analysis. John Wiley & Sons.[Google Scholar].

[fsn34000-bib-0006] Bu, S. Y. , Hunt, T. S. , & Smith, B. J. (2009). Dried plum polyphenols attenuate the detrimental effects of TNF‐α on osteoblast function coincident with up‐regulation of Runx2, Osterix and IGF‐I. The Journal of Nutritional Biochemistry, 20(1), 35–44. 18495459 10.1016/j.jnutbio.2007.11.012

[fsn34000-bib-0007] Chai, S. C. , Hooshmand, S. , Saadat, R. L. , Payton, M. E. , Brummel‐Smith, K. , & Arjmandi, B. H. (2012). Daily apple versus dried plum: Impact on cardiovascular disease risk factors in postmenopausal women. Journal of the Academy of Nutrition and Dietetics, 112(8), 1158–1168. 22818725 10.1016/j.jand.2012.05.005

[fsn34000-bib-0008] Chiu, H.‐F. , Huang, Y.‐C. , Lu, Y.‐Y. , Han, Y.‐C. , Shen, Y.‐C. , Golovinskaia, O. , Venkatakrishnan, K. , & Wang, C.‐K. (2017). Regulatory/modulatory effect of prune essence concentrate on intestinal function and blood lipids. Pharmaceutical Biology, 55(1), 974–979. 28164731 10.1080/13880209.2017.1285323 PMC6130511

[fsn34000-bib-0009] Clayton, Z. S. , Fusco, E. , Schreiber, L. , Carpenter, J. N. , Hooshmand, S. , Hong, M. Y. , & Kern, M. (2019). Snack selection influences glucose metabolism, antioxidant capacity and cholesterol in healthy overweight adults: A randomized parallel arm trial. Nutrition Research, 65, 89–98. 30952505 10.1016/j.nutres.2019.03.002 PMC7184323

[fsn34000-bib-0010] DerSimonian, R. , & Laird, N. (1986). Meta‐analysis in clinical trials. Controlled Clinical Trials, 7(3), 177–188. 3802833 10.1016/0197-2456(86)90046-2

[fsn34000-bib-0011] Donovan, J. L. , Meyer, A. S. , & Waterhouse, A. L. (1998). Phenolic composition and antioxidant activity of prunes and prune juice (*Prunus domestica*). Journal of Agricultural and Food Chemistry, 46(4), 1247–1252.

[fsn34000-bib-0012] Egger, M. , Smith, G. D. , Schneider, M. , & Minder, C. (1997). Bias in meta‐analysis detected by a simple, graphical test. BMJ, 315(7109), 629–634. 9310563 10.1136/bmj.315.7109.629 PMC2127453

[fsn34000-bib-0013] Foley, E. M. (2020). Alterations in the gut microbiome of Osteopenic men after three months of prune consumption. The Florida State University.

[fsn34000-bib-0014] Gallaher, C. M. , & Gallaher, D. D. (2008). Dried plums (prunes) reduce atherosclerosis lesion area in apolipoprotein E‐deficient mice. British Journal of Nutrition, 101(2), 233–239. 18761779 10.1017/S0007114508995684

[fsn34000-bib-0015] Godala, M. , Materek‐Kuśmierkiewicz, I. , Moczulski, D. , Rutkowski, M. , Szatko, F. , Gaszyńska, E. , Tokarski, S. , & Kowalski, J. (2017). The risk of plasma vitamin a, C, E and D deficiency in patients with metabolic syndrome: A case‐control study. Advances in Clinical and Experimental Medicine, 26(4), 581–586. 28691410 10.17219/acem/62453

[fsn34000-bib-0016] Guyatt, G. H. , Oxman, A. D. , Vist, G. E. , Kunz, R. , Falck‐Ytter, Y. , Alonso‐Coello, P. , & Schünemann, H. J. (2008). GRADE: An emerging consensus on rating quality of evidence and strength of recommendations. BMJ, 336(7650), 924–926. 18436948 10.1136/bmj.39489.470347.AD PMC2335261

[fsn34000-bib-0017] Hadi, A. , Askarpour, M. , Salamat, S. , Ghaedi, E. , Symonds, M. E. , & Miraghajani, M. (2020). Effect of flaxseed supplementation on lipid profile: An updated systematic review and dose‐response meta‐analysis of sixty‐two randomized controlled trials. Pharmacological Research, 152, 104622. 31899314 10.1016/j.phrs.2019.104622

[fsn34000-bib-0018] Higgins, J. P. , Altman, D. G. , Gøtzsche, P. C. , Jüni, P. , Moher, D. , Oxman, A. D. , Savovic, J. , Schulz, K. F. , Weeks, L. , Sterne, J. A. C. , Cochrane Bias Methods Group , & Cochrane Statistical Methods Group . (2011). The Cochrane Collaboration's tool for assessing risk of bias in randomised trials. BMJ, 343, d5928. 22008217 10.1136/bmj.d5928 PMC3196245

[fsn34000-bib-0019] Higgins, J. P. , & Thompson, S. G. (2002). Quantifying heterogeneity in a meta‐analysis. Statistics in Medicine, 21(11), 1539–1558. 12111919 10.1002/sim.1186

[fsn34000-bib-0020] Higgins, J. P. , Thompson, S. G. , Deeks, J. J. , & Altman, D. G. (2003). Measuring inconsistency in meta‐analyses. BMJ, 327(7414), 557–560. 12958120 10.1136/bmj.327.7414.557 PMC192859

[fsn34000-bib-0021] Hong, M. Y. , Kern, M. , Nakamichi‐Lee, M. , Abbaspour, N. , Ahouraei Far, A. , & Hooshmand, S. (2021). Dried plum consumption improves total cholesterol and antioxidant capacity and reduces inflammation in healthy postmenopausal women. Journal of Medicinal Food, 24(11), 1161–1168. 33978491 10.1089/jmf.2020.0142

[fsn34000-bib-0022] Howarth, L. , Petrisko, Y. , Furchner‐Evanson, A. , Nemoseck, T. , & Kern, M. (2010). Snack selection influences nutrient intake, triglycerides, and bowel habits of adult women: A pilot study. Journal of the American Dietetic Association, 110(9), 1322–1327. 20800123 10.1016/j.jada.2010.06.002

[fsn34000-bib-0023] Hozo, S. P. , Djulbegovic, B. , & Hozo, I. (2005). Estimating the mean and variance from the median, range, and the size of a sample. BMC Medical Research Methodology, 5(1), 1–10. 15840177 10.1186/1471-2288-5-13 PMC1097734

[fsn34000-bib-0024] Igwe, E. O. , & Charlton, K. E. (2016). A systematic review on the health effects of plums (*Prunus domestica* and *Prunus salicina*). Phytotherapy Research, 30(5), 701–731. 26992121 10.1002/ptr.5581

[fsn34000-bib-0025] Joseph, P. , Kutty, V. R. , Mohan, V. , Kumar, R. , Mony, P. , Vijayakumar, K. , Islam, S. , Iqbal, R. , Kazmi, K. , Rahman, O. , Yusuf, R. , Anjana, R. M. , Mohan, I. , Rangarajan, S. , Gupta, R. , & Yusuf, S. (2022). Cardiovascular disease, mortality, and their associations with modifiable risk factors in a multi‐national South Asia cohort: A PURE substudy. European Heart Journal, 43(30), 2831–2840. 35731159 10.1093/eurheartj/ehac249

[fsn34000-bib-0026] Kumar, A. , Hooshmand, S. , & Arjmandi, B. H. (2009). Dried plum polyphenols decreased markers of inflammation and lipid peroxidation in RAW264. 7 macrophage cells. The FASEB Journal, 23, 547.544.

[fsn34000-bib-0027] Lucas, E. A. , Juma, S. , Stoecker, B. J. , & Arjmandi, B. H. (2000). Prune suppresses ovariectomy‐induced hypercholesterolemia in rats. The Journal of Nutritional Biochemistry, 11(5), 255–259. 10876098 10.1016/s0955-2863(00)00073-5

[fsn34000-bib-0028] Minaiyan, M. , Ghannadi, A. , Movahedian, A. , Ramezanlou, P. , & Osooli, F. (2014). Effect of the hydroalcoholic extract and juice of Prunus divaricata fruit on blood glucose and serum lipids of normal and streptozotocin‐induced diabetic rats. Research in Pharmaceutical Sciences, 9(6), 421–429. 26339257 PMC4326980

[fsn34000-bib-0029] Mitchell, M. N. (2012). Interpreting and visualizing regression models using Stata (Vol. 558). Stata Press College Station.

[fsn34000-bib-0030] Moher, D. , Liberati, A. , Tetzlaff, J. , Altman, D. G. , & Group*, P . (2009). Preferred reporting items for systematic reviews and meta‐analyses: The PRISMA statement. Annals of Internal Medicine, 151(4), 264–269. 19622511 10.7326/0003-4819-151-4-200908180-00135

[fsn34000-bib-0031] Noratto, G. , Martino, H. S. , Simbo, S. , Byrne, D. , & Mertens‐Talcott, S. U. (2015). Consumption of polyphenol‐rich peach and plum juice prevents risk factors for obesity‐related metabolic disorders and cardiovascular disease in Zucker rats. The Journal of Nutritional Biochemistry, 26(6), 633–641. 25801980 10.1016/j.jnutbio.2014.12.014

[fsn34000-bib-0032] Potì, F. , Santi, D. , Spaggiari, G. , Zimetti, F. , & Zanotti, I. (2019). Polyphenol health effects on cardiovascular and neurodegenerative disorders: A review and meta‐analysis. International Journal of Molecular Sciences, 20(2), 351. 30654461 10.3390/ijms20020351 PMC6359281

[fsn34000-bib-0033] Santhakumar, A. B. , Kundur, A. R. , Sabapathy, S. , Stanley, R. , & Singh, I. (2015). The potential of anthocyanin‐rich queen garnet plum juice supplementation in alleviating thrombotic risk under induced oxidative stress conditions. Journal of Functional Foods, 14, 747–757.

[fsn34000-bib-0034] Shimizu, M. , Hashiguchi, M. , Shiga, T. , Tamura, H.‐o. , & Mochizuki, M. (2015). Meta‐analysis: Effects of probiotic supplementation on lipid profiles in normal to mildly hypercholesterolemic individuals. PLoS ONE, 10(10), e0139795. 26473340 10.1371/journal.pone.0139795 PMC4608827

[fsn34000-bib-0035] Stacewicz‐Sapuntzakis, M. (2013). Dried plums and their products: Composition and health effects–an updated review. Critical Reviews in Food Science and Nutrition, 53(12), 1277–1302. 24090144 10.1080/10408398.2011.563880

[fsn34000-bib-0036] Stacewicz‐Sapuntzakis, M. , Bowen, P. E. , Hussain, E. A. , Damayanti‐Wood, B. I. , & Farnsworth, N. R. (2001). Chemical composition and potential health effects of prunes: A functional food? Critical Reviews in Food Science and Nutrition, 41(4), 251–286. 11401245 10.1080/20014091091814

[fsn34000-bib-0037] Stewart, J. , McCallin, T. , Martinez, J. , Chacko, S. , & Yusuf, S. (2020). Hyperlipidemia. Pediatrics in Review, 41(8), 393–402. 32737252 10.1542/pir.2019-0053

[fsn34000-bib-0038] Sung, M.‐K. , Park, C.‐H. , Lee, E.‐J. , & Park, M.‐Y. (2005). Effects of plum and cherry intake on oxidative stress markers and blood lipid profiles in healthy subjects.

[fsn34000-bib-0039] Timmis, A. , Vardas, P. , Townsend, N. P. , Torbica, A. , Katus, H. , De Smedt, D. , Gale, C. P. , Maggioni, A. P. , Petersen, S. E. , Huculeci, R. , Kazakiewicz, D. , de Benito Rubio, V. , Ignatiuk, B. , Raisi‐Estabragh, Z. , Pawlak, A. , Karagiannidis, E. , Treskes, R. , Gaita, D. , Beltrame, J. F. , … Atlas Writing Group, European Society of Cardiology . (2022). European Society of Cardiology: Cardiovascular disease statistics 2021. European Heart Journal, 43(8), 716–799. 35016208 10.1093/eurheartj/ehab892

[fsn34000-bib-0040] Tinker, L. F. , Schneeman, B. O. , Davis, P. A. , Gallaher, D. D. , & Waggoner, C. R. (1991). Consumption of prunes as a source of dietary fiber in men with mild hypercholesterolemia. The American Journal of Clinical Nutrition, 53(5), 1259–1265. 1850578 10.1093/ajcn/53.5.1259

[fsn34000-bib-0041] Tobias, A. (1999). Assessing the influence of a single study in the meta‐analysis estimate. Stata Technical Bulletin, 47, 15–17.

[fsn34000-bib-0042] Trimurtini, I. , Ridwan, A. , Pontjosuda, F. , & Priyadi, H. (2021). The anti‐hypercholesterolemic effect of the ethanol extract of plum flesh in male rats of wistar strain induced by a high‐fat diet. Annals Of The Romanian Society For Cell Biology, 25(4),15237–15243.

[fsn34000-bib-0043] Tucakovic, L. , Colson, N. , Santhakumar, A. B. , Kundur, A. R. , Shuttleworth, M. , & Singh, I. (2018). The effects of anthocyanins on body weight and expression of adipocyte's hormones: Leptin and adiponectin. Journal of Functional Foods, 45, 173–180.

[fsn34000-bib-0044] Utsunomiya, H. , Yamakawa, T. , Kamei, J. , Kadonosono, K. , & Tanaka, S.‐I. (2005). Anti‐hyperglycemic effects of plum in a rat model of obesity and type 2 diabetes, Wistar fatty rat. Biomedical Research, 26(5), 193–200. 16295695 10.2220/biomedres.26.193

[fsn34000-bib-0045] Walkowiak‐Tomczak, D. (2008). Characteristics of plums as a raw material with valuable nutritive and dietary properties‐a review. Polish Journal Of Food And Nutrition Sciences, 58(4), 401–405.

[fsn34000-bib-0046] Walkowiak‐Tomczak, D. , Reguła, J. , & Śmidowicz, A. (2018). Effect of prune Prunus domestica consumption on blood lipid profile in patients with moderate hypercholesterolemia. Acta Scientiarum Polonorum. Hortorum Cultus, 17(6), 17–25.

[fsn34000-bib-0047] Wang, Z. , Lam, K. L. , Hu, J. , Ge, S. , Zhou, A. , Zheng, B. , Zeng, S. , & Lin, S. (2019). Chlorogenic acid alleviates obesity and modulates gut microbiota in high‐fat‐fed mice. Food Science & Nutrition, 7(2), 579–588. 30847137 10.1002/fsn3.868 PMC6392816

