# Supplementary material for: The effects of plum products consumption on lipid profile in adults: A systematic review and dose–response meta‐analysis
Source: Food Sci Nutr. 2024 Feb 25;12(5):3080–96. doi: 10.1002/fsn3.4000 (PMC11077222; doi:10.1002/fsn3.4000)
Supplement: Supplementary file 2 — File S2.. [file FSN3-12-3080-s002.docx]

Supplementary file :2Search strategy in PubMed database.

| pubmed |
| --- |
| (black plums[Title/Abstract] OR "eugenia jambolana"[Title/Abstract] OR "syzygium cumini"[Title/Abstract] OR jamun[Title/Abstract] OR plums[Title/Abstract] OR "prunus salcina"[Title/Abstract] OR "prunus damestica"[Title/Abstract] OR prunes[Title/Abstract] OR "prunes juice"[Title/Abstract] OR "plum tree"[Title/Abstract] OR "plum trees"[Title/Abstract] OR plum[Title/Abstract] OR plums[Title/Abstract] OR prunus cerasifera[Title/Abstract] OR "cherry plum tree"[Title/Abstract] OR "cherry plum trees"[Title/Abstract] OR "prunus salicina"[Title/Abstract] OR "japanese plum"[Title/Abstract] OR "japanese plums"[Title/Abstract]) AND (Intervention[Title/Abstract] OR "Intervention Study"[Title/Abstract] OR "Intervention Studies"[Title/Abstract] OR "controlled trial"[Title/Abstract] OR randomized[Title/Abstract] OR randomized[Title/Abstract] OR random[Title/Abstract] OR randomly[Title/Abstract] OR placebo[Title/Abstract] OR "clinical trial"[Title/Abstract] OR Trial[Title/Abstract] OR "randomized controlled trial"[Title/Abstract] OR "randomized clinical trial"[Title/Abstract] OR RCT[Title/Abstract] OR blinded[Title/Abstract] OR "double blind"[Title/Abstract] OR "double blinded"[Title/Abstract] OR trial[Title/Abstract] OR "clinical trial"[Title/Abstract] OR trials[Title/Abstract] OR "Pragmatic Clinical Trial"[Title/Abstract] OR "Cross-Over Studies"[Title/Abstract] OR "Cross-Over"[Title/Abstract] OR "Cross-Over Study"[Title/Abstract] OR parallel[Title/Abstract] OR "parallel study"[Title/Abstract] OR "parallel trial"[Title/Abstract]) |
